# Supplementary material for: Visualizing the ribonucleoprotein content of single bunyavirus virions reveals more efficient genome packaging in the arthropod host
Source: Commun Biol. 2021 Mar 22;4:345. doi: 10.1038/s42003-021-01821-y (PMC7985392; doi:10.1038/s42003-021-01821-y)
Supplement: Supplementary file 2 — Supplementary Information [file 42003_2021_1821_MOESM2_ESM.pdf]

Supplementary Information

Visualizing the ribonucleoprotein content of single bunyavirus virions reveals more efficient genome packaging in the arthropod host

Erick Bermúdez-Méndez, Eugene A. Katrukha, Cindy M. Spruit, Jeroen Kortekaas, Paul J. Wichgers Schreur

* Correspondence: paul.wichgersschreur@wur.nl

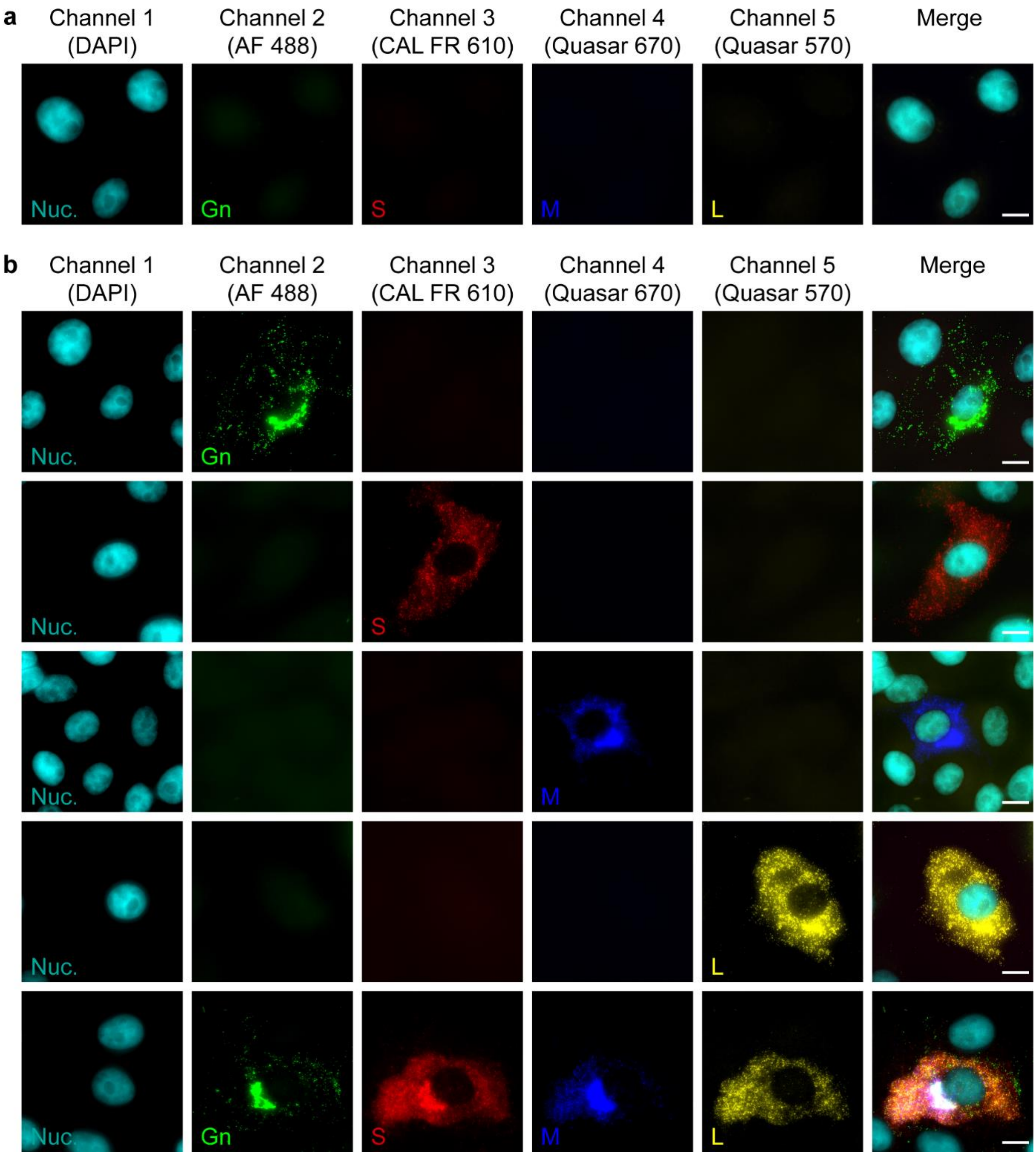

Supplementary Fig. 1 Specificity of RVFV FISH probe sets and antibodies. **a, b** Vero E6 cells were mock-infected (**a**) or infected with RVFV (MOI 0.75) (**b**) and cells were fixed at 8 h post-infection. The S segment (N gene; red), M segment (polyprotein gene; blue) and L segment (RdRp gene; yellow) were hybridized using probe sets labelled with CAL Fluor Red 610, Quasar 670 and Quasar 570, respectively. Progeny RVFV particles (green) were detected with antibody 4-D4[1] targeting the Gn glycoprotein in combination with Alexa Fluor 488-conjugated secondary antibodies. Cell nuclei (cyan) were visualized with DAPI. Mock-infected cells (**a**) were treated simultaneously with the three probe sets and antibodies. RVFV infected cells (**b**) were treated with either one probe set or antibodies at a time as single-color controls (first four rows) or simultaneously with the three probe sets and antibodies (last row). Scale bars, 10 µm.

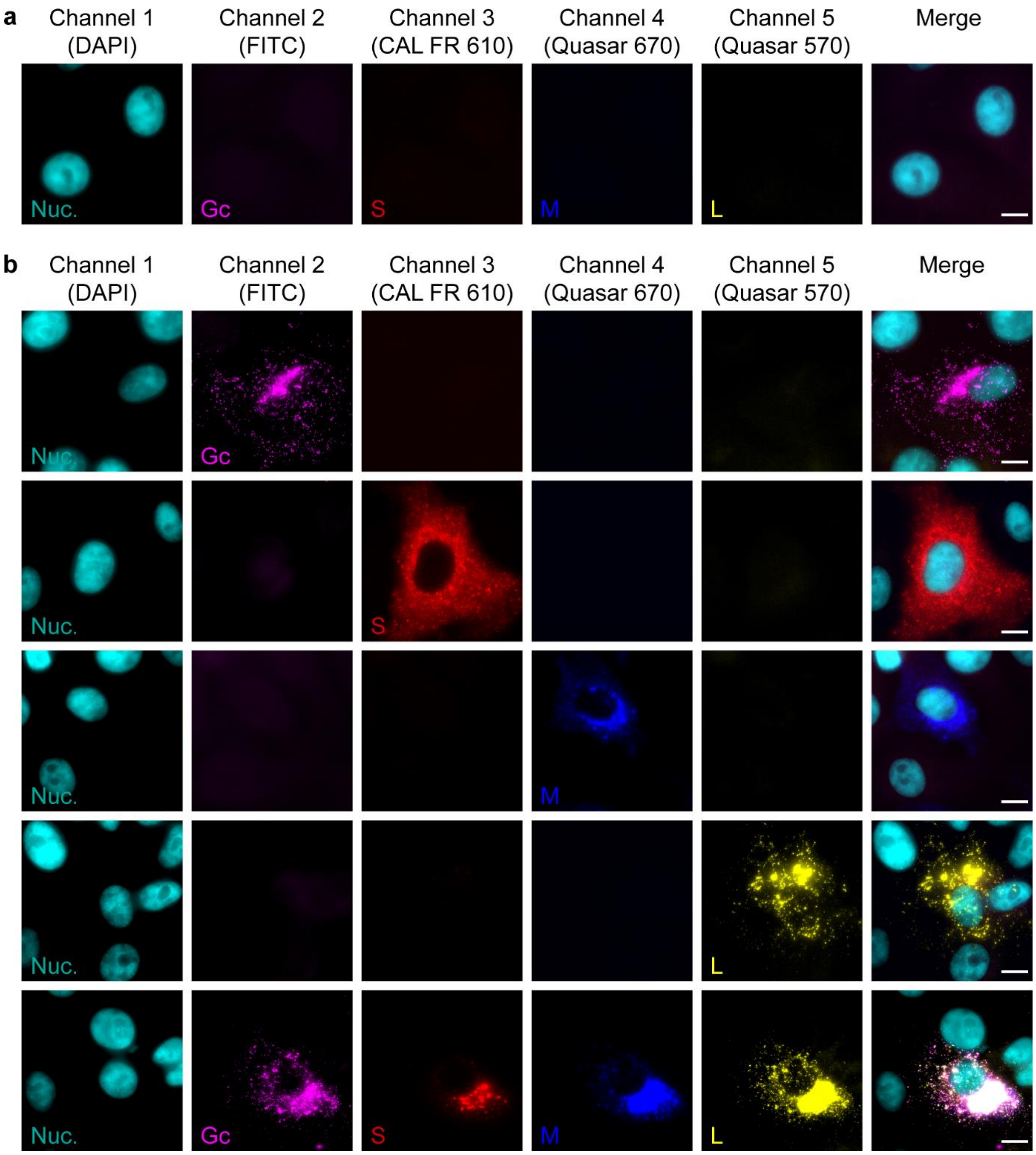

Supplementary Fig. 2 Specificity of SBV FISH probe sets and antibodies. **a, b** Vero E6 cells were mock-infected (**a**) or infected with SBV (MOI 0.33) (**b**) and cells were fixed at 8 h post-infection. The S segment (N gene; red), M segment (polyprotein gene; blue) and L segment (RdRp gene; yellow) were hybridized using probe sets labelled with CAL Fluor Red 610, Quasar 670 and Quasar 570, respectively. Progeny SBV particles (magenta) were detected with serum from an immunized rabbit[2] targeting the Gc glycoprotein in combination with FITC-conjugated secondary antibodies. Cell nuclei (cyan) were visualized with DAPI. Mock-infected cells (**a**) were treated simultaneously with the three probe sets and antibodies. SBV infected cells (**b**) were treated with either one probe set or antibodies at a time as single-color controls (first four rows) or simultaneously with the three probe sets and antibodies (last row). Scale bars, 10 µm.

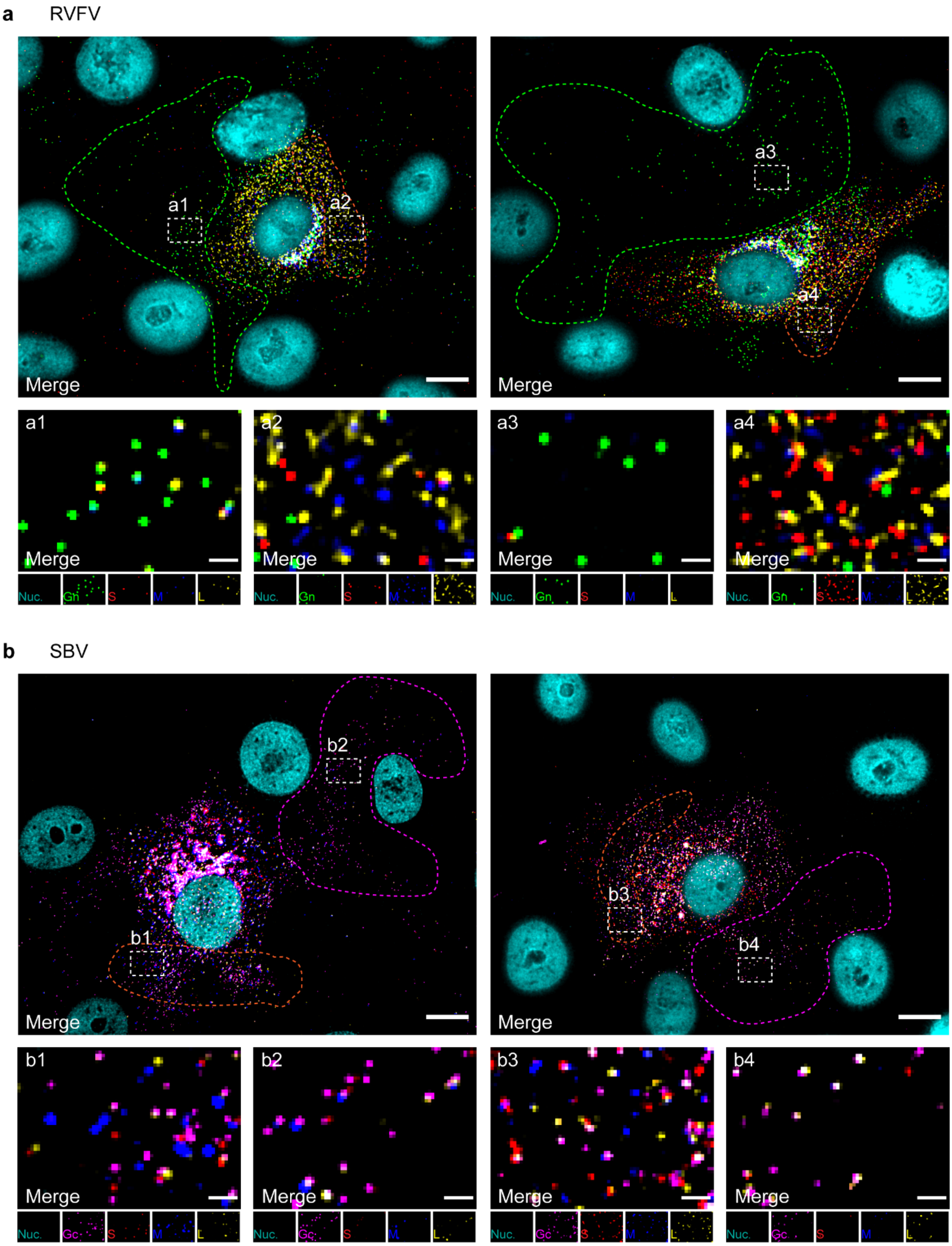

Supplementary Fig. 3 Representative regions of interest for the analysis of bunyavirus infected mammalian cells and their progeny virions using single-molecule vRNA FISH-immunofluorescence. **a, b** Vero E6 cells were infected with RVFV (MOI 0.50-0.75) (**a**) or SBV (MOI 0.33) (**b**) and cells were fixed at 8 h post-infection. The S segment (N gene; red), M segment (polyprotein gene; blue) and L segment (RdRp gene; yellow) were hybridized using probe sets labelled with CAL Fluor Red 610, Quasar 670 and Quasar 570, respectively. Progeny RVFV

particles (green) were detected with antibody 4-D4[1] targeting the Gn glycoprotein in combination with Alexa Fluor 488-conjugated secondary antibodies. Progeny SBV particles (magenta) were detected with serum from an immunized rabbit[2] targeting the Gc glycoprotein in combination with FITC-conjugated secondary antibodies. Cell nuclei (cyan) were visualized with DAPI. The orange dashed contours depict representative regions of interest selected for quantification of cytoplasmic vRNPs. The green (**a**) and magenta (**b**) dashed contours depict representative regions of interest selected for determining the genome composition of RVFV and SBV extracellular virions, respectively, through co-localization analysis. Middle rows of **a** and **b** show magnifications of smaller regions of interest within the dashed contours (indicated in top rows as white dashed boxes and labelled as a1-4 and b1-4). Bottom rows of **a** and **b** show individual channels of the magnified regions of interest. Main images are merged maximum intensity projections of five channels. Due to a higher fluorescence intensity of the green and magenta channels compared to the other channels, spots co-localizing with the glycoprotein may sometimes appear masked and not entirely evident in merged images. Scale bars, 10 µm (top rows) and 1 µm (middle rows).

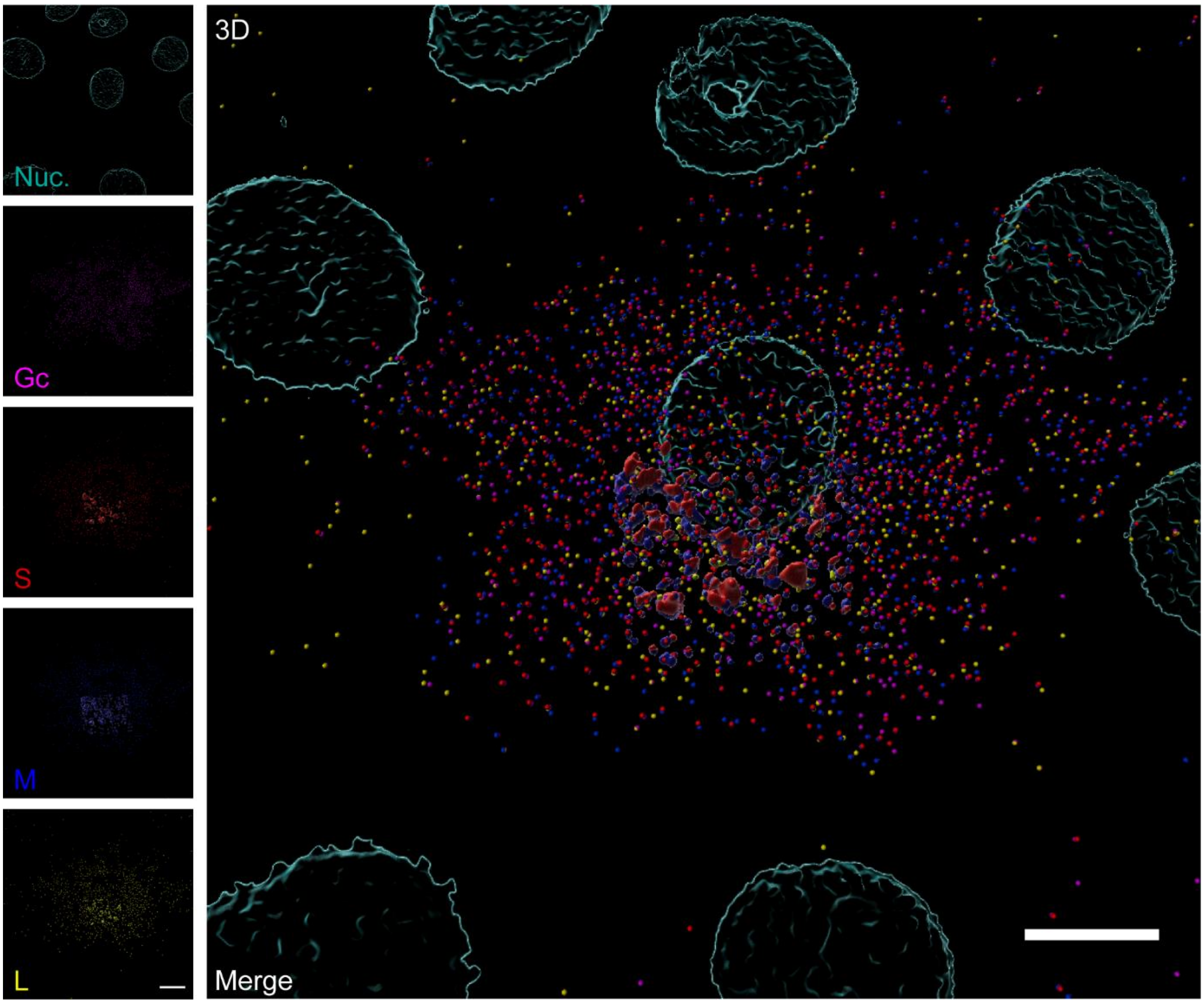

Supplementary Fig. 4 Single-molecule vRNA FISH-immunofluorescence of SBV infected mammalian cells. Vero E6 cells were infected with SBV (MOI 0.33) and cells were fixed at 8 h post-infection. The S segment (N gene; red), M segment (polyprotein gene; blue) and L segment (RdRp gene; yellow) were hybridized using probe sets labelled with CAL Fluor Red 610, Quasar 670 and Quasar 570, respectively. Progeny SBV particles were detected with serum from an immunized rabbit[2] targeting the Gc glycoprotein in combination with FITC-conjugated secondary antibodies. Cell nuclei (cyan) were visualized with DAPI. The three-dimensional representation showing the spatial distribution of vRNPs and virions was created with Imaris using the Surfaces and Spots modes. Accumulation of vRNPs in a perinuclear region shows active vRNP recruitment to the site of virion assembly. Co-localization of vRNPs and virions is depicted by merged spheres. Scale bars, 10 µm.

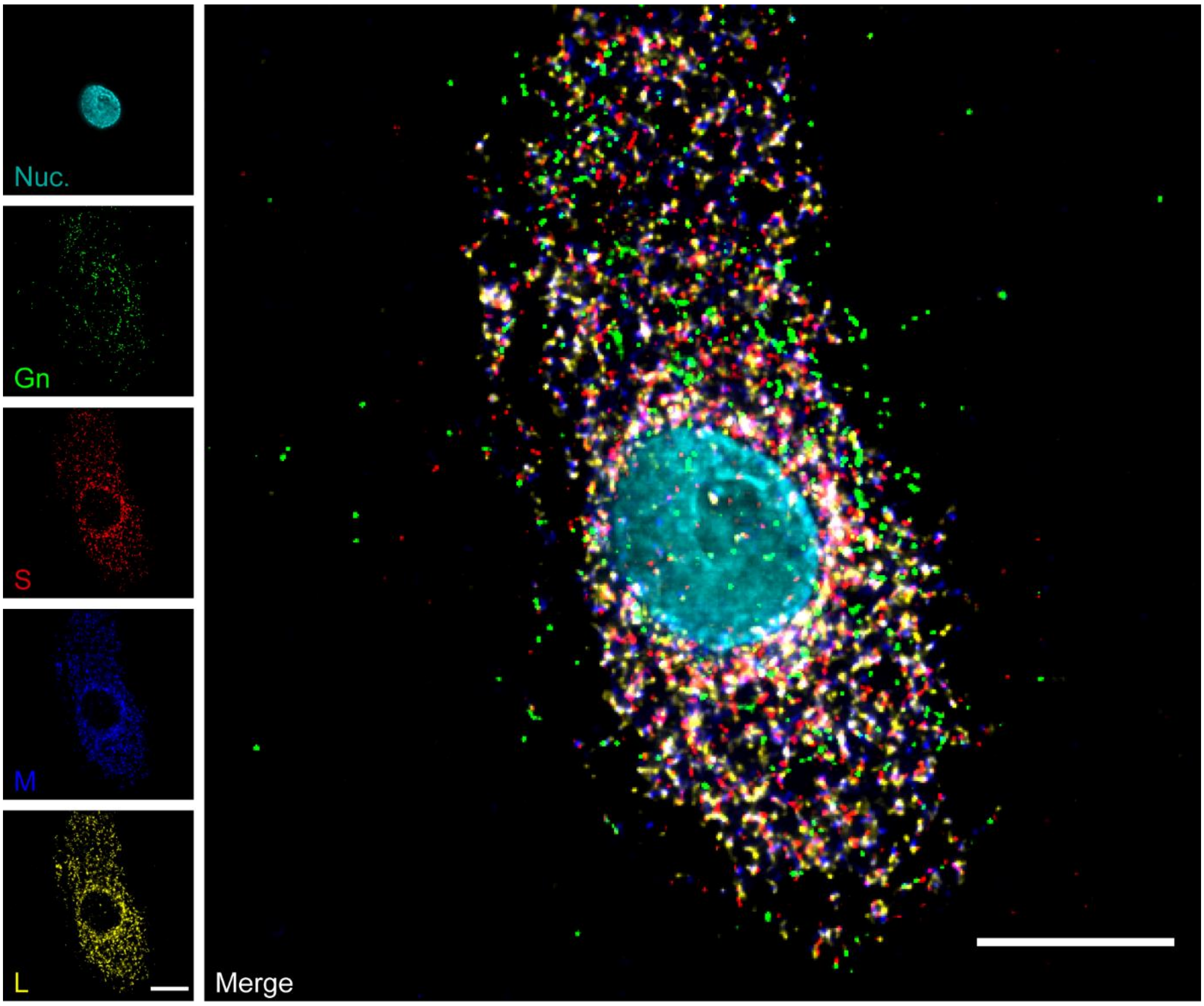

Supplementary Fig. 5 Single-molecule vRNA FISH-immunofluorescence of a RVFV infected insect cell. C6/36 cells were infected with RVFV (MOI 0.75) and cells were fixed at 32 h post-infection. The S segment (N gene; red), M segment (polyprotein gene; blue) and L segment (RdRp gene; yellow) were hybridized using probe sets labelled with CAL Fluor Red 610, Quasar 670 and Quasar 570, respectively. Progeny RVFV particles (green) were detected with antibody 4-D4[1] targeting the Gn glycoprotein in combination with Alexa Fluor 488-conjugated secondary antibodies. Cell nuclei (cyan) were visualized with DAPI. The main image merges maximum intensity projections of five channels (shown on the left). Due to a higher fluorescence intensity of the green channel compared to the other channels, spots co-localizing with the glycoprotein may sometimes appear masked and not entirely evident in merged images. Scale bars, 10 µm.

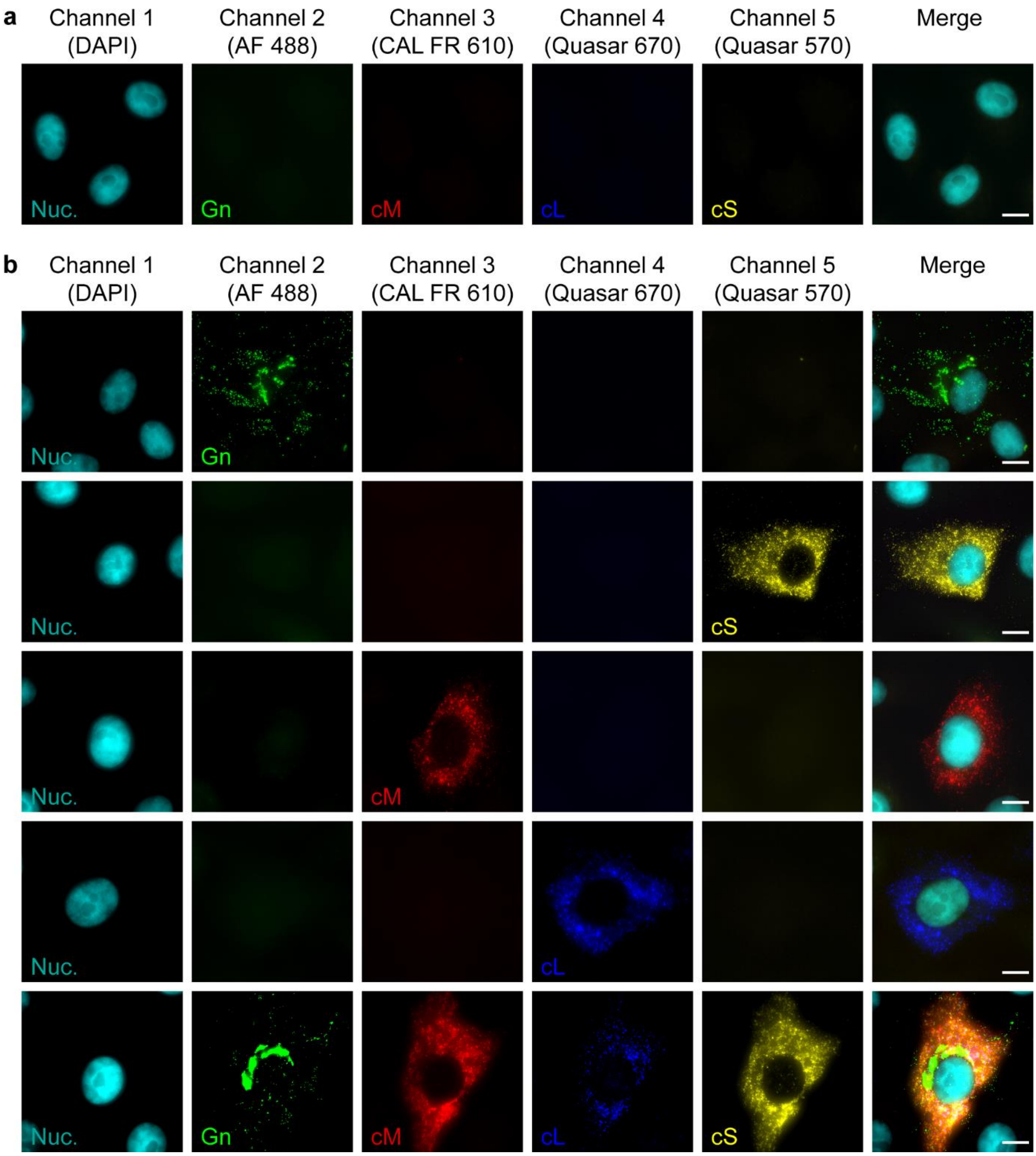

Supplementary Fig. 6 Specificity of RVFV complementary RNAs (cRNAs) FISH probe sets and antibodies. **a, b** Vero E6 cells were mock-infected (**a**) or infected with RVFV (MOI 0.75) (**b**) and cells were fixed at 8 h post-infection. The S segment cRNA (N gene; yellow), M segment cRNA (polyprotein gene; red) and L segment cRNA (RdRp gene; blue) were hybridized using probe sets labelled with Quasar 570, CAL Fluor Red 610, Quasar 670, respectively. Progeny RVFV particles (green) were detected with antibody 4-D4[1] targeting the Gn glycoprotein in combination with Alexa Fluor 488-conjugated secondary antibodies. Cell nuclei (cyan) were visualized with DAPI. Mock-infected cells (**a**) were treated simultaneously with the three probe sets and antibodies. RVFV infected cells (**b**) were treated with either one probe set or antibodies at a time as single-color controls (first four rows) or simultaneously with the three probe sets and antibodies (last row). Scale bars, 10 µm.

Supplementary Table 1 Primers for cDNA synthesis of viral genome segments.

Target	Name	Sequence
RVFV-Clone 13-S and RVFV-35/74-S	JR860-For	ACAAAGCTCCCTAGAGATACA
RVFV-Clone 13-M and RVFV-35/74-M	JR861-For	GACACAAAGACGGTGCATTA
RVFV-Clone 13-L and RVFV-35/74-L	JR890-For	GACACAAAGGCGCCCAATC
SBV-NL-F6-S	JR875-For	GTGAACTCCACTATTAACTACAGA
SBV-NL-F6-M	JR891-For	GAGTAGTGAACTACCACAATCAA
SBV-NL-F6-L	JR892-For	GTAGTGTACCCCTAATTACAATCAC

Supplementary Table 2 Primers for RT-qPCR amplifications of viral genome fragments.

Target	Name	Sequence
RVFV-Clone 13-S and RVFV-35/74-S	JR907-For	TCCAGTTTGCTGCTCAA
	JR908-Rev	CTGCTTTAAGAGTTCGATAACC
RVFV-Clone 13-M and RVFV-35/74-M	JR909-For	GCTGATGGCTTGAACAAC
	JR910-Rev	GTCTCTCACACCGAACTATC
RVFV-Clone 13-L and RVFV-35/74-L	JR911-For	TCGATAGATGTGGAAGATATGG
	JR912-Rev	CGTCATTCATCATGGGAAAC
SBV-NL-F6-S	JR878-For	CGGGTATGTGGCATTTATTG
	JR879-Rev	GACCATCTTGGCCTTCTT
SBV-NL-F6-M	JR882-For	CGACGTGGATTGAAGATAATG
	JR883-Rev	GAGGCTCTGTGAATTGTTAAG
SBV-NL-F6-L	JR886-For	CCCTGGATTGATGAGGATAC
	JR887-Rev	GACTCATGGAATGTCAGTTTAG

Supplementary References

1. Keegan, K. & Collett, M. S. Use of bacterial expression cloning to define the amino acid sequences of antigenic determinants on the G2 glycoprotein of Rift Valley fever virus. *Journal of Virology* **58**, 263–270 (1986).
2. Oymans, J. *et al.* Reverse Genetics System for Shuni Virus, an Emerging Orthobunyavirus with Zoonotic Potential. *Viruses* **12**, 455 (2020).
