# Supplementary material for: Visualizing the ribonucleoprotein content of single bunyavirus virions reveals more efficient genome packaging in the arthropod host
Source: Commun Biol. 2021 Mar 22;4:345. doi: 10.1038/s42003-021-01821-y (PMC7985392; doi:10.1038/s42003-021-01821-y)
Supplement: Supplementary file 3 — Description of Additional Supplementary Files [file 42003_2021_1821_MOESM3_ESM.pdf]

Description of Additional Supplementary Files

File name: Supplementary Movie 1

Description: Three-dimensional representation of individual virus particles released by a RVFV infected Vero E6 cell as shown in Fig. 2d. Progeny virions (green) were detected with antibody 4-D4 targeting the Gn glycoprotein in combination with Alexa Fluor 488-conjugated secondary antibodies. Cell nuclei (cyan) were visualized with DAPI. Gn accumulates in a perinuclear region, the site of virion assembly. The spatial distribution of virions and cell nuclei was created with Imaris using the Surfaces and Spots modes. Dynamic scale bar.

File name: Supplementary Movie 2

Description: Three-dimensional representation of individual virus particles released by a SBV infected Vero E6 cell as shown in Fig. 2e. Progeny virions (magenta) were detected with serum from an immunized rabbit targeting the Gc glycoprotein in combination with FITC-conjugated secondary antibodies. Cell nuclei (cyan) were visualized with DAPI. The spatial distribution of virions and cell nuclei was created with Imaris using the Surfaces and Spots modes. Dynamic scale bar.

File name: Supplementary Movie 3

Description: Three-dimensional representation of vRNPs and individual virus particles in a RVFV infected Vero E6 cell detected by single-molecule vRNA FISH-immunofluorescence as shown in Fig. 3c. S segment (N gene; red), M segment (polyprotein gene; blue), L segment (RdRp gene; yellow), progeny RVFV particles (green) and cell nuclei (cyan). Accumulation of vRNPs and co-localization to the same perinuclear region as Gn show active vRNP recruitment to the site of virion assembly. Co-localization of vRNPs and virions is depicted by merged spheres. The spatial distribution of virions and cell nuclei was created with Imaris using the Surfaces and Spots modes. Dynamic scale bar.

File name: Supplementary Movie 4

Description: Three-dimensional representation of vRNPs and individual virus particles in a SBV infected Vero E6 cell detected by single-molecule vRNA FISH-immunofluorescence as shown in Supplementary Fig. 4. S segment (N gene; red), M segment (polyprotein gene; blue), L segment (RdRp gene; yellow), progeny SBV particles (magenta) and cell nuclei (cyan). Accumulation of vRNPs in a perinuclear region shows active vRNP recruitment to the site of virion assembly. Co-localization of vRNPs and virions is depicted by merged spheres. The spatial distribution of virions and cell nuclei was created with Imaris using the Surfaces and Spots modes. Dynamic scale bar.

File name: Supplementary Data 1

Description: Oligonucleotide sequences of RNA FISH probe sets.

File name: Supplementary Data 2

Description: Source data underlying Figs. 1b-i, 1k-m, 2f-g, 4a-e, 5e-f and 6c.
