# Supplementary material for: Visualizing the ribonucleoprotein content of single bunyavirus virions reveals more efficient genome packaging in the arthropod host
Source: Commun Biol. 2021 Mar 22;4:345. doi: 10.1038/s42003-021-01821-y (PMC7985392; doi:10.1038/s42003-021-01821-y)
Supplement: Supplementary file 10 — Reporting Summary [file 42003_2021_1821_MOESM10_ESM.pdf]

nature research

Corresponding author(s): Paul J. Wichgers Schreur

Last updated by author(s): Jan 31, 2021

Reporting Summary

Nature Research wishes to improve the reproducibility of the work that we publish. This form provides structure for consistency and transparency in reporting. For further information on Nature Research policies, see our Editorial Policies and the Editorial Policy Checklist.

Statistics

For all statistical analyses, confirm that the following items are present in the figure legend, table legend, main text, or Methods section.

n/a	Confirmed	
☐	☒	The exact sample size (*n*) for each experimental group/condition, given as a discrete number and unit of measurement
☐	☒	A statement on whether measurements were taken from distinct samples or whether the same sample was measured repeatedly
☐	☒	The statistical test(s) used AND whether they are one- or two-sided<br>*Only common tests should be described solely by name; describe more complex techniques in the Methods section.*
☒	☐	A description of all covariates tested
☐	☒	A description of any assumptions or corrections, such as tests of normality and adjustment for multiple comparisons
☐	☒	A full description of the statistical parameters including central tendency (e.g. means) or other basic estimates (e.g. regression coefficient) AND variation (e.g. standard deviation) or associated estimates of uncertainty (e.g. confidence intervals)
☒	☐	For null hypothesis testing, the test statistic (e.g. *F*, *t*, *r*) with confidence intervals, effect sizes, degrees of freedom and *P* value noted<br>*Give P values as exact values whenever suitable.*
☒	☐	For Bayesian analysis, information on the choice of priors and Markov chain Monte Carlo settings
☒	☐	For hierarchical and complex designs, identification of the appropriate level for tests and full reporting of outcomes
☒	☐	Estimates of effect sizes (e.g. Cohen's *d*, Pearson's *r*), indicating how they were calculated

Our web collection on statistics for biologists contains articles on many of the points above.

Software and code

Policy information about availability of computer code

Data collection	RT-qPCR data were acquired with 7500 Fast System version 1.5.1. (Applied Biosystems).<br>Fluorescence microscopy images were acquired with ZEN 2.6 Pro (ZEISS, Germany).
Data analysis	Fluorescence microscopy images were z-aligned (if required) in ZEN 2.6 Pro (ZEISS, Germany), deconvolved using Huygens Professional version 19.10 (Scientific Volume Imaging B.V., The Netherlands).<br>3D data was converted to maximum intensity projections using Z-project within ImageJ. Detection, quantification and co-localization analyses of individual spots were performed in ImageJ in combination with the plugin ComDet version 0.5.0 (https://github.com/ekatrukha/ComDet).<br>Imaris 9.5 (Bitplane, Switzerland) was utilized to create optimal 3D representations of the data.<br>Statistical analysis was performed with Prism 8 (GraphPad).

For manuscripts utilizing custom algorithms or software that are central to the research but not yet described in published literature, software must be made available to editors and reviewers. We strongly encourage code deposition in a community repository (e.g. GitHub). See the Nature Research guidelines for submitting code & software for further information.

Data

Policy information about availability of data

All manuscripts must include a data availability statement. This statement should provide the following information, where applicable:

- Accession codes, unique identifiers, or web links for publicly available datasets
- A list of figures that have associated raw data
- A description of any restrictions on data availability

The authors declare that the data supporting the findings of this study are available within the paper and its supplementary information files. Source data

underlying Figs. 1b-i, 1k-m, 2f-g, 4a-e, 5e-f and 6c are provided in the Supplementary Data 2 file.

Field-specific reporting

Please select the one below that is the best fit for your research. If you are not sure, read the appropriate sections before making your selection.

[x] Life sciences [ ] Behavioural & social sciences [ ] Ecological, evolutionary & environmental sciences

For a reference copy of the document with all sections, see nature.com/documents/nr-reporting-summary-flat.pdf

Life sciences study design

All studies must disclose on these points even when the disclosure is negative.

Sample size	No statistical methods were used to predetermine the sample size.
Data exclusions	No data were excluded from the analyses. When a sample was below the limit of detection of a specific assay, this was stated in the figure legend. The criteria used to select images suitable for analysis is described within the manuscript.
Replication	When appropriate, the specific number of replicates and/or samples analyzed was stated.
Randomization	Randomization of the samples was not relevant for the purpose of the present study.
Blinding	Blinding was not relevant for the purpose of the present study.

Reporting for specific materials, systems and methods

We require information from authors about some types of materials, experimental systems and methods used in many studies. Here, indicate whether each material, system or method listed is relevant to your study. If you are not sure if a list item applies to your research, read the appropriate section before selecting a response.

Materials & experimental systems

n/a	Involved in the study	
[ ]	[x]	Antibodies
[ ]	[x]	Eukaryotic cell lines
[x]	[ ]	Palaeontology and archaeology
[ ]	[x]	Animals and other organisms
[x]	[ ]	Human research participants
[x]	[ ]	Clinical data
[x]	[ ]	Dual use research of concern

Methods

n/a	Involved in the study	
[x]	[ ]	ChIP-seq
[x]	[ ]	Flow cytometry
[x]	[ ]	MRI-based neuroimaging

Antibodies

Antibodies used	Hybridoma 4D-4 (Keegan, K. & Collett, M. S. Use of bacterial expression cloning to define the amino acid sequences of antigenic determinants on the G2 glycoprotein of Rift Valley fever virus. Journal of Virology 58, 263–270 (1986)).<br>Serum from an experimentally infected sheep (in-house).<br>Serum from an immunized rabbit (Oymans, J. et al. Reverse Genetics System for Shuni Virus, an Emerging Orthobunyavirus with Zoonotic Potential. Viruses 12, 455 (2020)).<br>HRP-conjugated rabbit polyclonal anti-mouse immunoglobulins (Dako)<br>HRP-conjugated rabbit polyclonal anti-sheep IgG (ab6747 Abcam)<br>Goat polyclonal anti-mouse IgG labelled with Alexa Fluor 488 (A-11001 Invitrogen)<br>Goat polyclonal anti-rabbit IgG labelled with FITC (sc-2012 Santa Cruz Biotechnology)
Validation	Commercial antibodies were validated as described by the manufacturers. The specificity of the antibodies used in smFISH-Immunofluorescence assays was validated experimentally. Data are presented in Supplementary Figs. 1, 2 and 6. Mock-infected samples and samples without primary antibodies were used as negative controls.

Eukaryotic cell lines

Policy information about cell lines

Cell line source(s)	Vero E6 and C6/36 cell lines were obtained from ATCC.<br>KC cells were kindly provided by Corinne Geertsema (Laboratory of Virology, Wageningen University)

Authentication	None of the cell lines used were authenticated.
Mycoplasma contamination	All cell lines were confirmed negative for mycoplasma.
Commonly misidentified lines (See ICLAC register)	Not applicable.

Animals and other organisms

Policy information about studies involving animals; ARRIVE guidelines recommended for reporting animal research

Laboratory animals	The study itself did not involve laboratory animals.<br>Plasma samples from an animal experiment within the scope of another study were obtained for analysis.
Wild animals	This study did not involve wild animals.
Field-collected samples	This study did not involve samples collected from the field.
Ethics oversight	The animal experiment within the scope of another study from which plasma samples were obtained for analysis was conducted in accordance with European regulations (EU directive 2010/63/EU) and the Dutch Law on Animal Experiments (Wod, ID number BWBR0003081). Permissions were granted by the Dutch Central Authority for Scientific Procedures on Animals (Permit Number: AVD4010020185564). Specific procedures were approved by the Animal Ethics Committees of Wageningen Research.

Note that full information on the approval of the study protocol must also be provided in the manuscript.
